# Chemical vs. mechanical microstructure evolution in drying colloid and polymer coatings

**DOI:** 10.1038/s41598-020-66875-0

**Published:** 2020-06-24

**Authors:** Thitiporn Kaewpetch, James F. Gilchrist

**Affiliations:** 1 Polymer Science and Engineering, Department of Materials Science and Engineering, Lehigh University, Bethlehem, PA USA; 2 Department of Chemical and Biomolecular Engineering, Lehigh University, Bethlehem, PA USA

**Keywords:** Soft materials, Colloids, Fluids, Gels and hydrogels

## Abstract

Colloidal based films have been widely developed for a wide range of applications including chemical and electrical barrier coatings, photonic materials, biomaterials, and pharmaceutical oral drug delivery. Many previous studies investigate methods to generate uniformity or desired stratification of the final components with a desired microstructure. Few studies have been able to investigate this microstructure *in*-*situ* during drying. This experimental study directly tracks fluorescent colloids that are either stable in suspension or have attractive interactions during the drying process using high speed laser scanning confocal microscopy to obtain details of microstructural evolution during drying. The colloidal microstructure in stable suspensions evolves continuously during drying. Microstructures in these systems have a signature Voronoi polyhedra distribution that is defined by lognormal curve having a constant standard deviation that only depends on its chemical composition. Those formulations having strongly attractive constituents have microstructure that is heterogeneous and non-monotonic due to the mechanics associated with internal convection and capillary forces. Toward the end of drying, the influence of the mode of microstructure rearrangements remains evident.

## Introduction

The colloquialism “watching paint dry” is synonymous with a process that seems unchanging except over long periods of time, or pseudo-steady state. The science of drying films, both a chemical and physical process, has a long history and ubiquitous application with regard to engineering and manufacturing surfaces with desired aesthetic and barrier properties^1–8^. More recently, thin films have been designed to have advanced chemical, photonic, and electrical properties for films that behave as sensors^9–13^, solar cells^14–16^, and LED^17–20^. In all of these applications, a uniform or prescribed distribution of the remaining non-volatile species that make up the resulting dry film is desirable to give reliable performance. However, there often remains a large separation between formulation and prediction of final film properties.

Previous work on drying thin films aimed to characterize the physics and structure of films that display stratification, nonuniformity, partitioning, or segregation of the components. Besides stratification, skin formation, a thin solid film at the air-film interface found in drying of latex having relatively low glass transition temperature, significantly reduces drying rate^21–23^. Scanning electron microscopy, including cryo-SEM, is most commonly used for imaging dissected samples after drying is complete^24–30^, though limited previous studies utilizing optical microscopy have given general information of stratification in the final film structure^31–33^. Partitioning during drying of colloidal suspensions drawn into thin films has been experimentally observed with limitations using scattering techniques^34,35^ or optical coherence tomography^36^ but it has predominantly been studied through simulations to understand the microstructural evolution. These studies generally concur that for stable suspensions the Péclet number, Pe = UL/D ≫ 1 for one of the constituents leads to partitioning, where U is the velocity of the air-film interface, L is related to the colloid diameter, and D is the colloid and/or polymer diffusivity. Cracking in these films during drying through hydrodynamic stress also imparts heterogeneity in the final film structure and alters its physical, optical, and chemical properties^37–41^.

What is perhaps missing from this body of literature is the microstructural evolution of the film constituents. Various approaches can be envisioned with the purpose of dictating the final structure through tuning the interparticle interactions in the formulation. In themselves, the structure of gels has received much fundamental numerical and experimental investigation to understand the relationship between structure and subtle differences in interparticle attraction^42–44^. These studies, with exceptions^45–47^, largely avoid the dominating macroscopic forces such as hydrodynamic drag from convection and capillary forces that can occur in drying films. These forces will dominate the structural evolution in drying films because surface energy will yield the gel microstructure for all systems except those at highest density and those having the strongest particle attractions, such as sintered ceramics.

This fundamental study aims to describe the key differences in the internal evolution of the microstructure between stable and strongly gelled samples. During drying of a colloid-polymer film, watching the film dry from the inside allows direct measurement of the particle microstructure during the hour-long drying process. These materials, by composition, most resemble thin films that can be used for oral drug delivery^48–51^, though these are simplified versions using only the basic ingredients that make up the primary film composition. This includes aqueous solutions that have dispersed polymer, polyethylene oxide, or PEO, and silica (SiO_2_) microspheres. The microspheres act as a surrogate for a hypothetical insoluble active ingredient. Citric acid is used to adjust the pH and stability of the dispersed phases. The purpose of this work is to show the differences in the evolution of microstructure in light of the inter-particle interactions.

## Results

**Figure 1:**
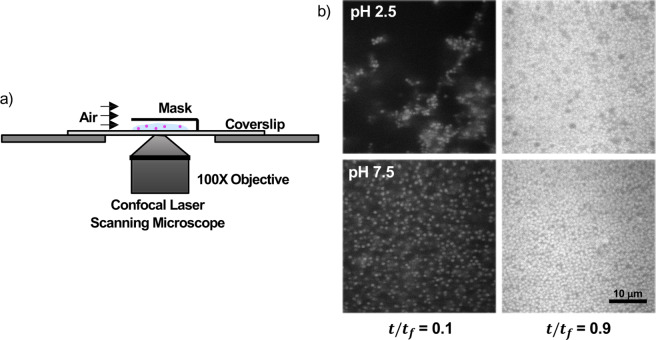
(**a**) Experimental setup. The blade coated sample is dried directly on the microscope under a mask that allows unidirectional drying. (**b**) Representative scans 5 μm above the substrate early and late in drying.

The general approach of this study is straightforward. After tuning the particle interactions of these formulations through adjustment in pH, as characterized by the solution rheology, direct imaging of the drying film was performed. The suspension contained 0.860 ± 0.020 μm core-shell synthesized fluorescent SiO_2_ microspheres^52,53^ with volume fraction of *ϕ*_0_ = 0.05 and 200,000 g/mol MW PEO solutions with weight fraction of *w*_*PEO*_ = 0.05 or 0.10. After pH adjustment to pH 2.5 or pH 7.5, the sample was blade coated creating a film with the initial thickness of 200 μm and then partially masked to drive drying from one direction to another, similar to continuous processing conditions (Fig. 1). Drying occurred over roughly an hour at ambient temperature of 21 °C and relative humidity of 20–50%. Below, the details for each of these steps are outlined.

### Rheology of PEO and microsphere-PEO suspensions

**Figure 2:**
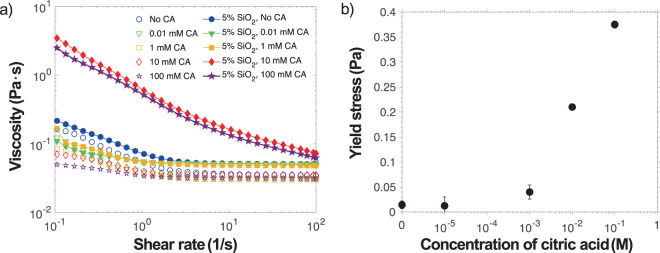
Rheology of the solutions including (**a**) viscosity as a function of shear rate of aqueous PEO (open symbols) and SiO_2_ microsphere-PEO solutions (closed symbols) with added citric acid 0 ≤ *C*_*ca*_ ≤ 0.1 M. Solutions having no added citric acid have pH of 7.5 and those at C_*ca*_ = 0.1 M have pH of 2.5. Concentrations in each sample are *w*_*PEO*_ = 0.05 and microsphere volume fraction of *ϕ*_0_ = 0.05. Yield stress of SiO_2_ microsphere-PEO solutions (**b**) increases significantly with sufficient added citric acid.

Rheology was performed on the coating formulations prior to drying to demonstrate the effect of added SiO_2_ microspheres and pH on the interaction of SiO_2_ and PEO. As shown in Fig. 2, all samples have shear thinning rheology. All solutions of PEO in water at varying citric acid concentrations *C*_*ca*_ show a modest degree of shear thinning, plateauing at a constant viscosity above a shear rate *γ *> 0.3 1/s. As expected, the zero shear viscosity decreases with increasing *C*_*ca*_ as the solution becomes a worse solvent for PEO.

Addition of SiO_2_ microspheres into these solutions demonstrates the opposite trend in zero shear viscosity with added citric acid than that found in purely polymer solutions. At neutral conditions, addition of microspheres uniformly increases the viscosity at all shear rates by approximately 0.01 Pa∙s, as is expected when there are only hydrodynamic interactions between the microspheres and PEO. This is unsurprising knowing bare SiO_2_ has a moderate negative charge at neutral pH and an isoelectric point ranging from pH values of 1.5 to 3^54^. The surface charge is reduced significantly by protonation at lower pH up until its isoelectric point. Here, a pH ~2.5 corresponds to *C*_*ca*_ = 100 mM. Addition of citric acid for *C*_*ca*_ ≤ 1 mM has a minor effect on the rheology. For samples where *C*_*ca*_ ≥ 10 mM, the viscosity increases by more than an order of magnitude. This suggests a strong interaction between PEO and SiO_2_, more than that of PEO responding to poor solvent quality as the pH is lowered. This adsorption is likely a result of the change in SiO_2_ surface charges and the nondissociated silanol groups on SiO_2_ surface hydrogen bonding with PEO. This association likely results in a heteroaggregated gel forming a network of PEO and the SiO_2_ microspheres.

In terms of formulation, besides simply increasing the viscosity, a significant yield stress forms for samples where *C*_*ca*_ ≥ 10 mM, shown in Fig. 2b. This yield stress is also indicative of the heteroaggregated gel network. For samples at rest, this yield stress can “lock in” the microstructure resisting the effects of gravitational sedimentation and Brownian motion of particles. As evaporation occurs, densification must occur increasing the local concentration of particles due to the overwhelming strength of the capillary force from the top air-film interface.

These suspensions are coated and then partially masked to drive nearly unidirectional drying to mimic the conditions in continuous roll-to-roll oven driers as opposed to drying of sessile droplets from all edges inward. During active coating of these samples, hydrodynamic deformation of the microstructure dominates for all samples. In heteroaggregated samples, their microstructure is destroyed and reforms quickly when flow is arrested. The shear rate during coating is greater than 10^2^ 1/s. After the coating process is complete and drying begins, all samples show evidence of lateral drift velocity at the early stages of drying (see ESI). Thin films having significant yield stress no longer demonstrate any macroscopic flow after this initial stage while those with weak or no yield stress continue to flow due to the evaporation flux. Even though lateral flow is primarily arrested, the microstructure evolves significantly during drying.

### Microstructure evolution during drying

**Figure 3:**
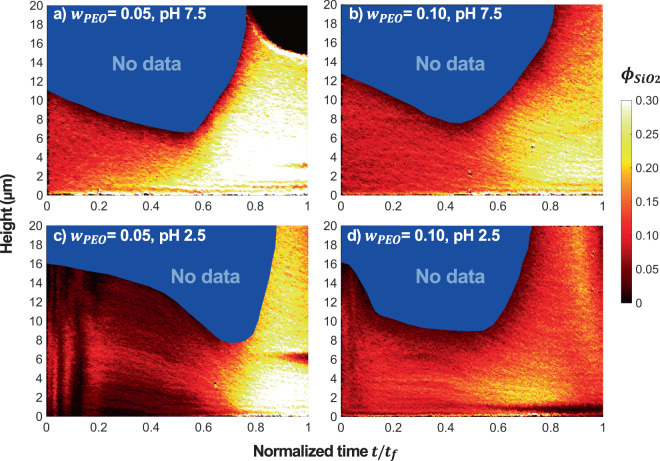
Concentration profile of SiO_2_ microspheres in SiO_2_-PEO mixtures as a function of normalized drying time *t/t*_*f*_. The mixture has initial SiO_2_ volume fraction *ϕ*_0_ = 0.05 and PEO weight fraction (**a**) *w*_*PEO*_ = 0.05 at pH 7.5, (**b**) *w*_*PEO*_ = 0.10 at pH 7.5, (**c**) *w*_*PEO*_ = 0.05 at pH 2.5, and (**d**) *w*_*PEO*_ = 0.10 at pH 2.5. Height indicates a distance from the substrate. A change in SiO_2_ volume fraction, *ϕ*_*SiO*2_, during drying is represented as a colour scale. The depth of reduced confidence in particle location due to a mismatch of refractive index is indicated in blue.

One obvious change that occurs in thin films during drying is the evolution of concentration as solvent flux from the top surface occurs during evaporation. This has been a challenge that has only been addressed through simulations^24,55–57^ and scattering experiments^34^. Through the benefit of tracking fluorescently labelled SiO_2_ microspheres using high speed confocal microscopy, the evolution in concentration of microspheres, *ϕ*_*SiO*2_, and their microstructure can be directly determined. Figure 3 shows this transient evolution as a spatio-temporal graph. The concentration profile near the substrate is shown as a function of drying time normalized by the total time for drying up until the arrest of the microstructure, *t*_*f*_ ~1 h, for all samples. The sample dries further after this time without microstructure changes. The evolution of concentration presents the challenge of variation of the index of refraction over time, limiting the depth to which accurate imaging can be obtained. The depth of reduced confidence in particle location is indicated in blue. This region shows diminishing ability to see through the suspension in the early stages of drying, and then better index matching as the polymer concentration increases.

In samples at pH 7.5, a stable suspension that begins without a yield stress and lower viscosity, the evolution of the concentration profile is relatively uniform. As solvent evaporates, the top interface moves toward the substrate, as seen by the clear air-film interface and lack of any particles above this region. At the lower formulated concentration of polymer, Fig. 3a, early in the drying process only a slight inhomogeneity in concentration occurs from 0 ≤ *t*/*t*_*f*_ ≤ 0.6. At *t*/*t*_*f*_ > 0.8, the top interface enters the observable region for this sample. Due to a difference in density of microspheres and polymer solution, the microspheres may be settling during drying. However, the maximum Péclet number for drying is Pe_D_ ~*O*(10) which is 3 order of magnitude larger than that for sedimentation, Pe_S_ ~*O*(10^−2^) (calculation is provided in the ESI). This indicates that the settling rate is slower than the advancing interface, causing the accumulation of particles near the interface. It is possible this partitioning occurs as a result of diffusiophoresis^41^. As predicted in prior studies, a higher concentration of particles, often referred to as a skin, is observed. Toward the end of this scan, some finer microstructure is observable as particle packing become denser and confined by the substrate. The particles do not crystalize with long range order, perhaps due to the presence of polymer and eventual particle destabilization as the concentration of polymer increases. Figure 3b shows a similar trend with less apparent gravitational settling due to the increased viscosity. This sample does not allow visualization of the top interface due to a greater degree of polymer resulting in a thicker final layer as the film completes drying. Within this viewable region, over many individual experiments, the resulting concentration is relatively homogenous. Inhomogeneity likely occurs over much larger length scales from the internal capillary-driven flow and certainly near edges of the film.

In samples at pH 2.5 where the film initially has a significant yield stress and higher viscosity from the SiO_2_-polymer heteroaggregation prior to drying, the space-time plot shows significant heterogeneity during drying. The profiles of samples shown are representative of several trials, each showing its own unique concentration evolution. In Fig. 3c, large variations of local concentration are observable at the beginning of drying. This results of large structural rearrangements of gel undergoing deformation from capillary-driven flow. Between 0.6 ≤ *t*/*t*_*f*_ ≤ 0.8, a relatively sudden increase in concentration occurs as the interface provides enough mechanical strength to compress the microstructure. After this point, the microsphere distribution is relatively uniform with the exception of local phase separation due to the internal stress developed in the drying film, seen at 6 μm at the end of this scan. Figure 3d, where the film also starts with a yield stress and the polymer concentration is double that of Fig. 3c, shows similar microstructural rearrangements early in the drying process. The final density of this film is not as high as other samples as the polymer fills in the voids of the remaining microsphere-polymer heteroaggregated microstructure.

**Figure 4:**
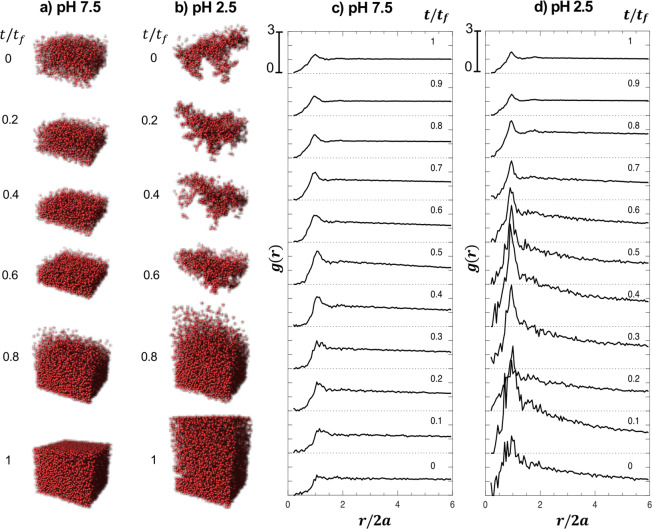
Rendered microstructure of observed particles through the drying process for (**a**) pH 7.5 and (**b**) pH 2.5 at arbitrary t/t_f_. The average spatial distribution of particles is indicated as radial distribution function, g(r), during drying suspensions of *𝜙*_0_ = 0.05 and *w*_*PEO*_ = 0.05 at (**c**) pH 7.5 and (**d**) pH 2.5, as a function of normalized separation distance r/2a, where a is a particle radius.

To highlight the apparent difference in microstructure evolution, Fig. 4a,b shows 3D rendered images for films having initial concentration w_PEO_ = 0.05 and pH 7.5 and 2.5 taken from movies of the microstructure evolution, included as Electronic Supplemental Material (ESI). Note these renderings are obtained from the experimentally determined particle locations from laser scanning confocal microscopy. From the very beginning of drying the microstructure is starkly different. At pH 7.5, microspheres are mostly uniformly distributed throughout the volume scanned and increase in density. At pH 2.5, the structure varies drastically in the early stages of drying. As noted in the concentration profiles shown in Fig. 3, uniformity dominates microstructures for both pH samples for *t*/*t*_*f*_ > 0.8, yet the microstructure for pH 2.5 is more open reflecting the lower average density of microstructures toward the end of drying.

The true advantage of having precise particle locations of the microspheres in these experiments is the ability to go beyond simply rendering the microstructure to demonstrate the statistical data underlying this microstructure, similar to that provided by simulations. A common tool for showing microstructural differences in suspensions is calculating the radial distribution function, g(r), as shown in Fig. 4c,d. At pH 7.5, there is no SiO_2_-PEO heteroaggregation and SiO_2_ microspheres are stabilized by their surface charge. The radial distribution function is relatively flat for *t*/*t*_*f*_ ≤ 0.3. Through increasing density during drying, particles at higher concentration demonstrate a peak at r/2a = 1 as is common in particle suspensions. This first peak in the microsphere structure also may result from depletion interactions induced from the concentrated polymer as drying proceeds. The small changes in g(r) occur gradually through drying. At pH 2.5, the gel that gives the initial static yield stress of this suspension results in attractions clearly shown in a broad peak at r/2a = 1. The broadness of this peak for *t*/*t*_*f*_ ≤ 0.6 results both from lower statistics associated with fewer particles in this open network, but also from the strong heteroaggregated gel^58^. Moreover, a long-range g(r) gradually approaches 1 from 2 < r/2a < 6 referring to the formation of open fractal structures^59^. For 0.6 < *t*/*t*_*f*_ < 0.8, the microspheres rapidly approach a density that results in a compact structure due to the evaporation and capillary compression from the interface, leading to the clear g(r) peak at r/2a = 1. This microstructure change occurs suddenly and relatively late in the drying process.

**Figure 5:**
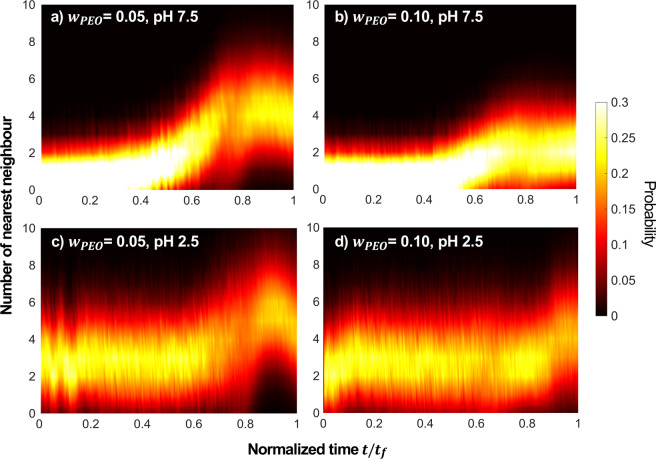
Distribution of neighbours in contact, *N*_*contact*_, of SiO_2_ during drying. The samples were prepared at *ϕ*_0_ = 0.05 and (**a**) *w*_*PEO*_ = 0.05 at pH 7.5, (**b**) *w*_*PEO*_ = 0.10 at pH 7.5, (**c**) *w*_*PEO*_ = 0.05 at pH 2.5, and (**d**) *w*_*PEO*_ = 0.10 at pH 2.5. Probability of number of *N*_*contact*_ is indicated in a colour bar.

The g(r) gives the average spatial distribution of particles without much detail regarding the particle-level heterogeneity. Unlike g(r) where the nearest neighbour is found pair-wise, the number of neighbours in contact, *N*_*contact*_, is used to determine the number of microspheres that are locally forming the network attached to a given particle (Fig. 5). As is done in prior figures, a common comparison between representative samples at pH 7.5 and pH 2.5 highlights the effect of attraction in the microstructure of these films during drying. The probability distribution of nearest neighbours is given from black to white through red.

At pH 7.5, repulsive interactions between SiO_2_ particles lead to a low *N*_*contact*_ when *t*/*t*_*f*_ ≤ 0.5 (Fig. 5a,b). *N*_*contact*_ still varies from 0 to 2 particles because of the random distribution of microspheres within the liquid, increasing as the microsphere density increases during drying. This distribution stays low until approximately half way through drying. For the *w*_*PEO*_ = 0.05 the average increases to approximately 4 particles with a wide distribution. This increase may signal the point of destabilization of the microspheres. For *w*_*PEO*_ = 0.10, the *N*_*contact*_ also increases and its distribution broadens later in drying, but plateaus with an average of approximately 2. It is perhaps the high polymer concentration and viscosity that arrests the motion of microspheres. In contrast, at pH 2.5 particles are arrested in contact at the beginning of the experiment. *N*_*contact*_ has an average of 3 microspheres and a large distribution ranging from 1 to 6 particles for *t*/*t*_*f*_ ≤ 0.5 (Fig. 5c,d). The dynamic rearrangement of the gel is apparent from the drastic fluctuations in the profile as the gel breaks and reorders during drying, after which the particle rearrangements occur at a kinematically slow rate. These fluctuations are primarily a result of the microscopic sampling of different gel structures rather than the local rearrangement of the microstructure. As is clear from the movies and Fig. 4a,b, the stress on the microstructure is far from one that creates primary particles that reorganize. For these gels, *N*_*contact*_ increases later in the drying profile than samples at pH 7.5.

The story told by the evolution in concentration, radial distribution function, and neighbours in contact suggests differences between microstructure of liquids and gels that are relatively unsurprising. As concentration increases, particles gradually come together. Upon closer inspection, this process is not gradual for gel samples at pH 2.5. After large fluctuations in microstructure because of internal flow and large-scale heterogeneity, the structure is relatively unchanged for a large part of the drying process, here apparent from roughly 0.2 ≤ *t*/*t*_*f*_ ≤ 0.7.

**Figure 6:**
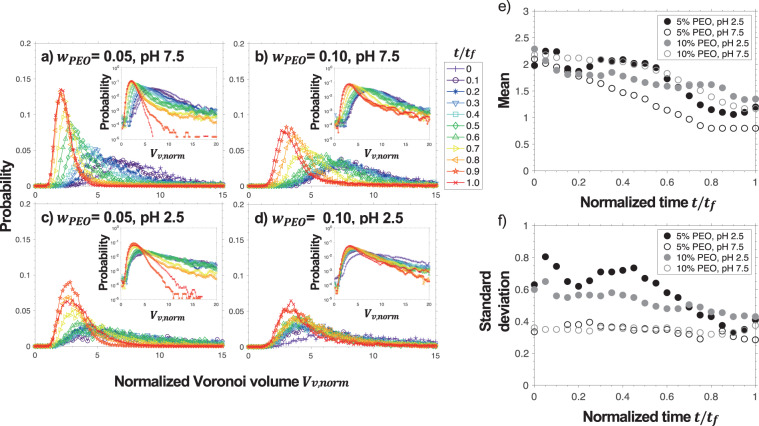
Normalized Voronoi volume, *V*_*v,norm*_, distribution of SiO_2_ in the SiO_2_-PEO mixtures while drying, 0 < *t/t*_*f*_ < 1. The mixtures consist of *ϕ*_0_ = 0.05 and (**a**) *w*_*PEO*_ = 0.05 at pH 7.5, (**b**) *w*_*PEO*_ = 0.10 at pH 7.5, (**c**) *w*_*PEO*_ = 0.05 at pH 2.5, and (**d**) *w*_*PEO*_ = 0.10 at pH 2.5. Insets show the same data plotted as a log-linear plot which is best fitted with lognormal distribution function, giving fitting parameters (**e**) mean, μ, and (**f**) standard deviation, σ, as a function of normalized drying time *t/t*_*f*_.

The signature of this relatively abrupt change in microstructure is highlighted in the Voronoi volume distribution shown in Fig. 6. The Voronoi volume, ***V***_*v*,*norm*_, for a given particle is the equivalent space closer to that particle than any other particles. It is normalized by the volume for each particle in a FCC maximum packing density of same sized microspheres, or ${V}_{FCC}=4{a}^{3}\surd 2$. Each inset in Fig. 6a–d represents the same data plotted as a log-linear plot to highlight the tail of the distribution. Each Voronoi distribution can be fitted to a lognormal distribution^60,61^

$$P=\frac{\alpha }{{V}_{v,norm}\sigma \sqrt{2\pi }}\,\exp \,\left(-\frac{{(\mathrm{ln}({V}_{v,norm})-\mu )}^{2}}{2{\sigma }^{2}}\right),$$

where *μ* and *σ* are the lognormal distribution mean and standard deviation, respectively. The magnitude,* α*, is used as a fitting parameter recognizing the experimental data cannot have a distribution that spans to infinity. The mean and standard deviations for these curves are plotted as a function of time in Fig. 6e,f.

In a general drying process, the distribution of *V*_*v*,*norm*_ is broad at *t*/*t*_*f*_ = 0 and becomes narrower at *t*/*t*_*f*_ = 1. Also, the distribution peak shifts from a larger to a smaller *V*_*v*,*norm*_ toward the limit of *V*_*v*,*norm*_ = 1 when *t*/*t*_*f*_ →1. For *w*_*PEO*_ = 0.05 and *w*_*PEO*_ = 0.10 at pH 7.5, this transition is continuous and monotonic as drying proceeds. In terms of their lognormal distributions, while the mean decreases monotonically, the standard deviation is constant and the same for both experiments throughout drying. This suggests that at each time during the experiment, the structure is similar to what would be expected if a sample was synthesized with those components with apparently no dependence on the history of drying. For this reason, we refer to microstructure evolution in stable suspensions solely as a “chemical process” where the structure is independent of the drying profile and only depends on the local concentration.

In contrast, those samples at pH 2.5 show non-monotonic evolution in their *V*_*v*,*norm*_ distribution. In Fig. 6e,f this discontinuous evolution is apparent. On examination of the fitted curves to these profiles, *μ* and *σ* are non-monotonic. This non-monotonic behavior suggests the microstructural rearrangements are largely a mechanical process of yielding and rearrangement. In Fig. 6f, for most of the time during drying *σ* is much larger for pH 2.5 than pH 7.5 where the particles have attractive interactions resulting in a larger degree of structural heterogeneity. This varying distribution in *σ* is much more apparent in the lower viscosity systems having initial *w*_*PEO*_ = 0.05 than for *w*_*PEO*_ = 0.10. It is unclear whether it is a result of the capillary number, *Ca* = *ηU*/*γ*, having larger value for higher degree of polymer loading, therefore resisting the effects of surface tension in rearranging the microstructure or if the interactions between particles are different for different polymer concentrations.

## Conclusions

While there are limitations in this approach, the data shown here definitively demonstrates two drastically different modes of microstructural rearrangements that depend on the particle-particle interactions that can be generalized past pH-dependent interactions. In designing films with engineered microstructures, a chemical route where the microstructure emulates that of a relatively isotropic distribution of particles gives a more predictable path. Alternatively, attractive particle-particle interactions set a microstructure that is immobilized at the smallest length scales for longer into the drying process until mechanical stresses dominate. This may be advantageous depending on the desired optical/mechanical/electromagnetic properties that depend on percolation or other microstructural characteristics. Clearly, challenges remain in balancing capillary forces, drying rate and internal microstructural stresses to engineer well-behaved systems. As an approach for predicting these behaviours, the capability offered in *in*-*situ* monitoring of the evolving microstructure will not be easily surpassed by simulations nor sacrificial techniques used previously.

## Methods

### Rheological measurements

Non-fluorescent SiO_2_ microspheres (Fuso Chemical Co, Japan) with 0.951 ± 0.022 μm diameter and 1.8 g/cm^3^ density were dispersed in a solution of Poly(ethylene oxide) or PEO (Colorcon®) with molecular weight of 200,000 g/mol and approximate density of 1.1 g/cm^3^ ^62^ to obtain the suspension of initial SiO_2_ volume fraction *ϕ*_0_ = 0 or 0.05, and PEO weight fraction *w*_*PEO*_ = 0.05. Before performing the rheological measurements, pH of the samples was adjusted. Viscosity and yield stress were determined using an Ares 2000 ex rheometer (TA Instruments) with a 0.29° 60 mm cone and plate geometry. Both viscosity and yield stress measurements were performed using a steady shear stress sweep method at 25 °C and within a shear rate range of 0.1 to 100 1/s.

### 2D and 3D microstructure evolution imaging

Core-shell Rhodamine B fluorescent SiO_2_ microspheres were synthesized following established protocols^52,53^ and characterized by using scanning electron microscopy. The synthesized fluorescent microsphere has a diameter of 0.860 ± 0.02 μm and approximate particle density of 1.8 g/cm^3^. The rhodamine B dye has an absorption wavelength of 543 nm and an emission wavelength of 580 nm. The dispersion of the fluorescent labelled microspheres in a 200,000 g/mol MW PEO solution were formulated at *ϕ*_0_ = 0.05 and *w*_*PEO*_ = 0.05 or 0.10. All formulations were adjusted to pH 2.5 or pH 7.5 before performing the experiment. The sample was blade-coated on a glass substrate and dried unidirectionally at ambient temperature of 21 °C and relative humidity of 20–50%. The initial film thickness is roughly 200 μm. The film microstructure was visualized in both two and three dimensions using high speed laser scanning confocal microscopy (Vteye, Visitech International) with a laser wavelength of 488 nm with a 510 long pass filter at 72 fps within 30 s time interval until a drying process completed. Total drying time was roughly 1–2 hours. Due to mismatch of refractive index, n, between silica microspheres (n_SiO2_ = 1.42) and PEO solutions (n_PEO_ = 1.45 and n_water_ = 1.33), the depth of confidence in particle location is limited, indicated in blue in Fig. 3.

### Image analysis

Location of fluorescent SiO_2_ microspheres in 2D and 3D scans were analysed using particle tracking algorithms developed by Crocker and Grier^63^ and additional 3D particle tracking algorithms provided by Professor Eric Weeks. A bandpass filter was applied before particle locations were identified from the brightest particle centroids^63^. From 2D scans, mean square displacement (MSD) was analyzed and correlated to particle diffusivity^64,65^. In case of 3D scans, a concentration profile of the SiO_2_ microspheres was generated from tracked positions as a function of normalized drying time *t*/*t*_*f*_. An algorithm of 3D radial distribution function, g(r), was used to obtain the g(r) analysis at different drying times. Distribution of neighbours in contact were also generated by an algorithm provided by Professor Eric Weeks. Noted that a maximum bond length parameter, b_max_, in the code is different for the sample at pH 2.5 (b_max_ = 1.3) and pH 7.5 (b_max_ = 1.1) due to the existence of PEO absorption on microsphere surface. Lastly, three-dimension Voronoi volume analysis was performed^66^.

## Supplementary information

Supplementary information.

Supplementary Movie S1.

Supplementary Movie S2.
