# Supplementary material for: Chemical vs. mechanical microstructure evolution in drying colloid and polymer coatings
Source: Sci Rep. 2020 Jun 24;10:10264. doi: 10.1038/s41598-020-66875-0 (PMC7314827; doi:10.1038/s41598-020-66875-0)
Supplement: Supplementary file 1 — Supplementary information. [file 41598_2020_66875_MOESM1_ESM.pdf]

Supplementary Information

Chemical vs. mechanical microstructure evolution in drying colloid and polymer coatings

Thitiporn Kaewpetch[a] and James F. Gilchrist[a,b]*

[a] Polymer Science and Engineering, Department of Materials Science and Engineering, Lehigh University, Bethlehem, PA, USA.
[b] Department of Chemical and Biomolecular Engineering, Lehigh University, Bethlehem, PA, USA.

1. Lateral velocity drift

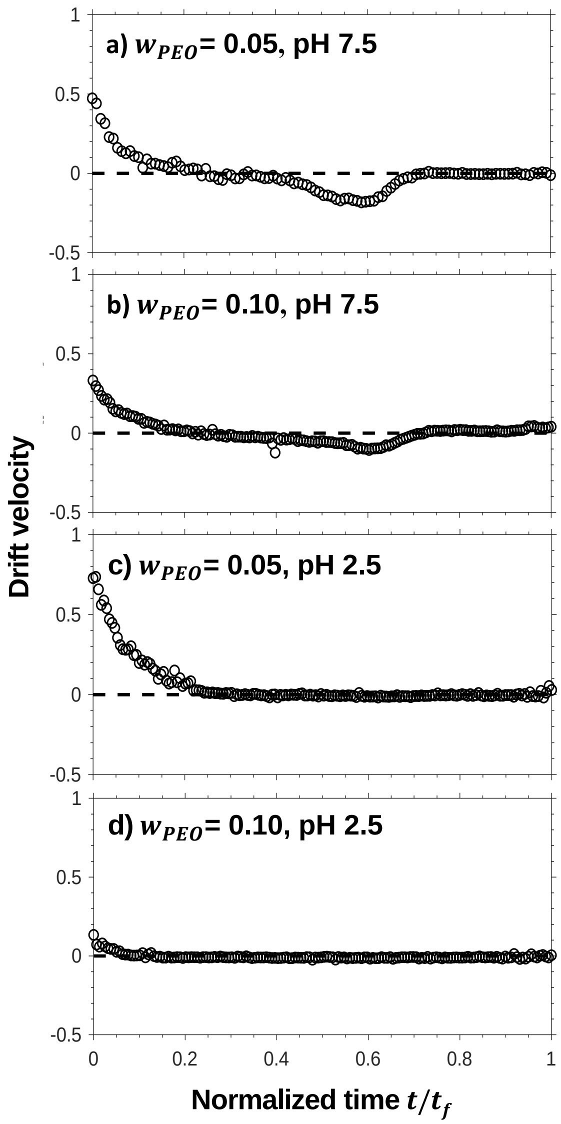

Figure S1. Lateral drift velocity during drying the samples at ϕ_0 = 0.05 and a) w_{PEO} = 0.05 at pH 7.5, b) w_{PEO} = 0.10 at pH 7.5, c) w_{PEO} = 0.05 at pH 2.5, and d) w_{PEO} = 0.10 at pH 2.5.

2. Péclet number

From 2D multiple particle tracking (MPT), particle trajectories are used to calculate mean square displacement (MSD)[1,2] ,

$$< \Delta r^2(t) > = < \Delta x^2(t) > + < \Delta y^2(t) > \quad (1)$$

where t is time, Δx is x-displacement, and Δy is y-displacement. It can be correlated to particle diffusivity, D,

$$< \Delta r^2(t) > = 2dD\tau^{\alpha} \quad (2)$$

where d is the number of dimension, τ is lag time, and α is logarithmic slope of the MSD. In this case, MSD and α during drying the suspension of ϕ_0 = 0.05 and w_{PEO} = 0.05 at pH 7.5. Only MSD and α at t/t_f = 0 are considered because this is the highest observed diffusivity. From equation (2) the diffusivity of the particle at t/t_f = 0 is 0.011 μm^2/s.

During drying thin film Péclet number, Pe_D, used to determine film formation is defined as

$$Pe_D = \frac{U_E H_0}{D} \quad (4)$$

while the sedimentation Pe_S[3] is

$$Pe_S = \frac{U_S H}{D} \quad (5)$$

where U_E is velocity of the interface that depends on evaporation rate, U_S is Stokes settling velocity of a spherical particle, H is initial film thickness, and D is particle diffusivity. This gives Pe_D of 20 and Pe_{sed} of 0.025.

3. Movies of the microstructure evolution

Two movies of the microstructure evolution are provided as supplementary information. One is the movie of microstructure changes in drying a suspension of ϕ_0 = 0.05 and w_{PEO} = 0.05 at pH 2.5 and another is the movie of microstructure changes during drying the sample of ϕ_0 = 0.05 and w_{PEO} = 0.05 at pH 7.5. These movies are obtained from experimentally tracked particle locations from laser scanning confocal microscopy.

4. Log-normal fits

Linear-log plots of Voronoi volume distribution during drying are best fitted with log-normal distribution above probability of 0.005. These fits provide two parameters, one is mean, μ, and another is standard deviation, σ.

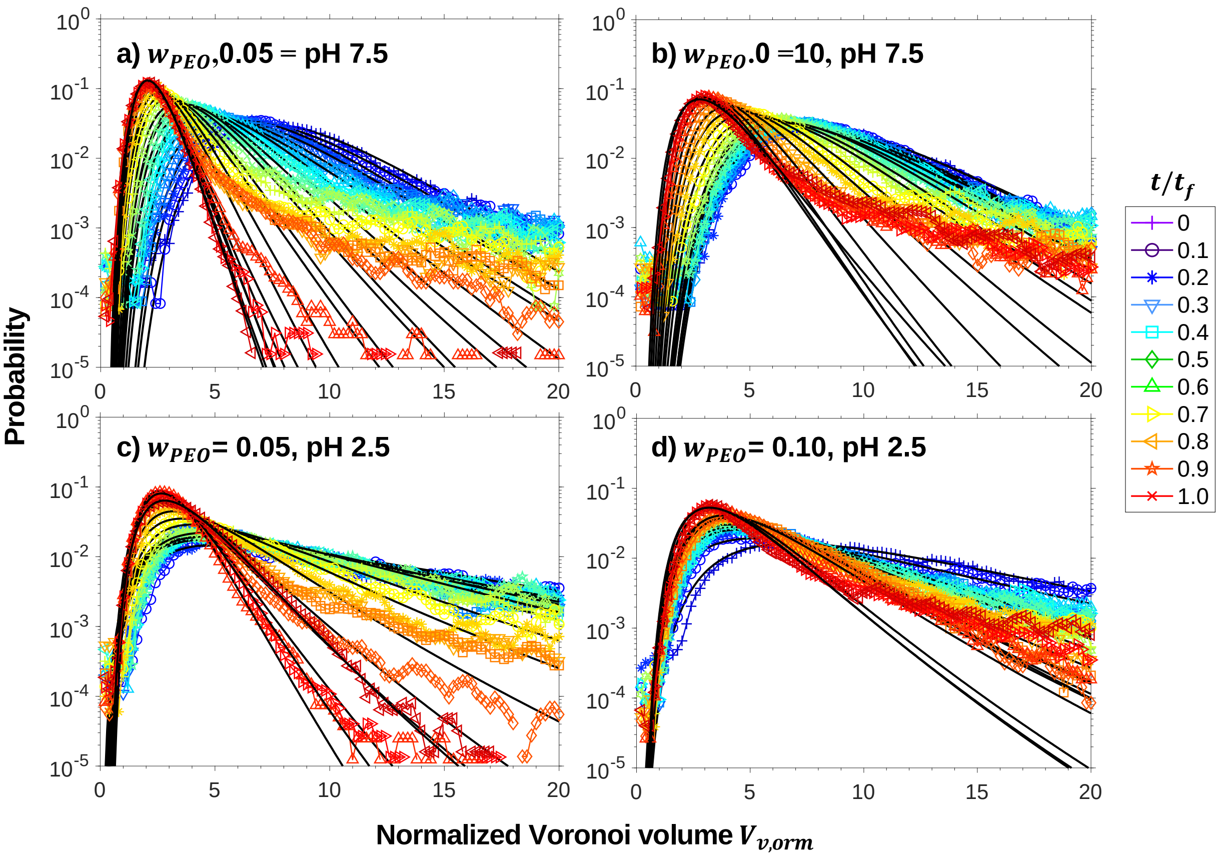

Figure S2. Log-normal fit on log-linear plot of Voronoi volume distribution. The samples initially contain ϕ_0 = 0.05 and a) w_{PEO} = 0.05 at pH 7.5, b) w_{PEO} = 0.10 at pH 7.5, c) w_{PEO} = 0.05 at pH 2.5, and d) w_{PEO} = 0.10 at pH 2.5. All fitting curves are agreed well with the sample data above the probability of 0.005.

References

S1 T. G. Mason, *Rheol. Acta*, 2000, **39**, 371–378.

S2 M. D. Wehrman, S. Lindberg and K. M. Schultz, *Soft Matter*, 2018, **14**, 5811–5820.

S3 C. M. Cardinal, Y. D. Jung, K. H. Ahn and L. F. Francis, *AIChE J.*, 2010, **56**, 2769–2780.
